# Postprandial inflammation across the aging spectrum

**DOI:** 10.1016/j.jnha.2024.100468

**Published:** 2025-01-08

**Authors:** Bryant H. Keirns, Natalie G. Keirns, Christina M. Sciarrillo, Austin R. Medlin, Sarah E. Fruit, Sam R. Emerson

**Affiliations:** a Department of Nutrition and Health Science, Ball State University, Muncie, 47306 IN, United States; b Department of Nutritional Sciences, Oklahoma State University, Stillwater, 74075 OK, United States; c Department of Health and Wellness Design, Indiana University School of Public Health, Bloomington, 47405 IN, United States

**Keywords:** Postprandial inflammation, Aging, Cardiovascular disease

## Abstract

**Objectives:**

Postprandial inflammation post-high-fat meals may be linked to cardiovascular disease (CVD). CVD incidence increases with age; however, whether older adults experience greater postprandial inflammation remains unclear. We examined whether analyzing age categorically versus continuously influenced relationships between age and postprandial inflammatory measures.

**Design:**

Cross-sectional.

**Setting:**

Laboratory for Applied Nutrition and Exercise Science at Oklahoma State University (Stillwater, OK, USA).

**Participants:**

56 apparently healthy adults ages 20–69 years.

**Measurements:**

We measured interleukin (IL)-1β, IL-6, IL-8, IL-10, and tumor necrosis factor (TNF)-α at baseline, 2-, 4-, and 6 -hs post-high-fat meal (9 kcal/kg; 70% fat). Data were examined in the full sample with paired t-tests (baseline to peak), by pre-defined age groups (i.e., 18–35, 36–49, 50–59, 60–69) using ANCOVA, and continuously using linear regression.

**Results:**

Across the full sample, IL-1β, IL-6, and IL-8 increased after the high-fat meal (*p*’s≤0.018). Cytokine differences post-high-fat meal by age category were generally not observed. However, age was positively associated with IL-6 incremental AUC when examined continuously (*b* = 0.029; *p* = 0.010).

**Conclusion:**

These data suggest increasing age is linked to a greater IL-6 response to a high-fat meal. Further, examining age continuously may have greater utility when studying aging and postprandial inflammation.

**Registration:**

N/A (secondary analysis)

## Highlights

• High-fat meal consumption increased IL-6, IL-8, and IL-1β in the full sample.
• Examined categorically, postprandial cytokines and age were largely unrelated.
• Examined continuously, postprandial IL-6 was positively associated with age.

## 1 Introduction

Repeated increases in inflammatory cytokines following high-fat meal consumption have been proposed as one mechanism linking Western-style diets to increased cardiovascular disease (CVD) risk [1,2]. Indeed, higher interleukin (IL)-6 is consistently reported in generally healthy adults 18–60 years following high-fat meals [3]. Additionally, changes in other cytokines that would be expected to promote an overall pro-inflammatory state (i.e., increased IL-1β, IL-8, tumor necrosis factor (TNF)-α) after high-fat meals have been reported in some cases in young and middle-aged populations [3]. However, despite many studies examining changes in postprandial cytokines, few have examined potential differences in younger versus older adults after high-fat meal intake.

CVD incidence increases with age, which is likely attributable to several factors, including higher basal indicators of inflammation (e.g., IL-6, TNF-α) [4]. However, whether more pronounced inflammation after high-fat meals is linked to older age – potentially contributing to CVD risk – has been relatively understudied. Specifically, two studies have observed that: (1) active participants ≥60 years had lower serum IL-10 and IL-23 post-high-fat meal compared to active participants 18–35 years [5], and (2) circulating IL-6, IL-1β, TNF-α, C-reactive protein, and monocyte chemoattractant protein-1 were not different following a high-fat meal in individuals 60–75 versus 20–25 years [6]. In addition to results being mixed, these studies have been relatively small (n = 6–8/group) and have used distinct age-groups, rather than examined age as a continuous spectrum. For these reasons, the goal of this work was to examine markers of postprandial inflammation in a larger sample across a broad age spectrum both within categorical age groups and continuously.

## 2 Methods

### 2.1 Overview

The present report is a secondary analysis of a study examining the validity and reliability of an abbreviated fat tolerance test across the aging spectrum (*unpublished*). Participants were apparently healthy and free of established cardiometabolic conditions (e.g., cardiovascular disease, type 2 diabetes), inflammatory conditions (e.g., rheumatoid arthritis, inflammatory bowel disease), and/or active illnesses. Briefly, all participants completed two “standard fat tolerances tests” (triglycerides measured hourly for six hours from an intravenous catheter [IV]; stayed in laboratory) and two “abbreviated fat tolerance tests” (triglycerides measured at baseline and four hours via single venipunctures). For the primary study, participants were recruited into four age categories (n = 15–16/group): 18–35 years, 36–49 years, 50–59 years, and 60–69 years. During the majority of participants’ first standard fat tolerance test, additional whole blood was collected at baseline, 2-, 4 -hs, and 6 -hs and stored as serum per standard procedures (i.e., allowed to clot for ∼30 min, centrifuged for 15 min at 2500 RPM at 4 °C, stored at −80 °C). However, due to technical difficulties with IVs (i.e., unable to set IV, malfunctions mid-session), complete serum sets were obtained for 56/60 participants (n = 11–16/group). These complete serum sets from the first standard fat tolerance tests were used to measure serum inflammatory markers in this study. All participants provided written informed consent and this project was carried out in accordance with the Declaration of Helsinki/ approved by the Oklahoma State Institutional Review Board.

### 2.2 Fat tolerance test

Prior to the fat tolerance test, participants avoided exercise for 24 -hs and consumed the same snack (peanut butter crackers; 200 kcal) before initiating a 10 -h overnight fast. Upon arrival, an IV was inserted into an antecubital vein using a 21-gauge needle and slow drip of 0.9% NaCl was initiated. After a baseline blood sample was collected, participants consumed a high-fat meal scaled to body weight (9 kcal/kg; 70% fat, 20% carbohydrate, 10% protein) consisting of blended unsweetened coconut cream, chocolate syrup, and pea protein powder. During the six-hour session, participants were allowed water ad libitum, but were not allowed to consume any other food or drink.

### 2.3 Serum analyses

Fasting metabolic parameters (i.e., cholesterol metrics, triglycerides, glucose, liver enzymes) were measured using a Piccolo Xpress clinical chemistry analyzer (Abbott) with lipid panel plus reagent discs. IL-1β, IL-6, IL-8, IL-10 and TNF-α were measured externally with a custom high-sensitivity bioplex (Eve Technologies) at baseline and 2-, 4-, and 6 -hs following the high-fat meal.

### 2.4 Body composition assessment

Body composition was assessed using whole-body dual-energy X-ray absorptiometry (DXA) scans. Additionally, trained research personnel measured waist circumference using a Gulick tape measurer.

### 2.5 Statistical analyses

All data were checked for normality (Shapiro-Wilk test), linearity (scatterplots of standardized residuals against predicted values), and outliers (*Z* = ± 3.3) prior to analyses. Outliers that impacted normality were excluded; otherwise, outliers were retained. One outlier was excluded for each outcome (i.e., IL-1β, IL-6, IL-8, IL-10 and TNF-α) and all outcomes were log-transformed prior to analyses to further correct for non-normality. When normality concerns persisted following transformation, alternative analytic approaches were employed as described below.

To confirm the expected changes in inflammatory markers to the high-fat meal, we first conducted paired-samples *t*-tests to assess changes in cytokine levels (i.e., IL-1β, IL-6, IL-8, IL-10 and TNF-α) from fasting to peak postprandial concentrations within the full sample. We also assessed potential differences in fasting, peak, change from baseline to peak (delta), and incremental area under the curve (AUC_i_) [7] for each cytokine by our predefined categorical age groups (18–35, 36–49, 50–59, and 60–69 years) using ANCOVAs (adjusted for sex and BMI) followed by Bonferroni’s *post hoc* test. Negative AUC_i_’s indicate a summative decrease from baseline for a given cytokine. Groups sharing the same letter (a or b) are not statistically different from one another based on *post hoc* analyses following ANCOVAs. For outcomes not meeting normality assumptions, linear regressions with age as a multicategorical predictor variable (reference: 18–35 years) and the same covariates were conducted in place of ANCOVAs. Lastly, our primary moderation analyses were conducted via linear regression to assess the continuous relationship between age and postprandial inflammation (i.e., AUC_i_) for each cytokine. All regression models included BMI as a potential moderating variable and sex as a covariate. All analyses were conducted in SPSS Statistics Version 27. Moderation analyses were conducted using the PROCESS macro version 3.5.3. [8]

## 3 Results

Participant characteristics can be found in Table A1. In the full sample, IL-1β (*M* = +0.47 ± 1.42 pg/mL, pre/post *p* = 0.018), IL-6 (*M* = +1.83 ± 2.86 pg/mL, pre/post *p* < 0.001), and IL-8 (*M* = +2.08 ± 4.47 pg/mL, pre/post *p* = 0.001) rose after the high-fat meal (all found in Table 1). No postprandial changes were observed for IL-10 or TNF-α across all participants (*p*’s ≥ 0.541; Table 1). When examining inflammatory markers by age category (i.e., 18–35, 36–49, 50–59, and 60–69 years; Table 1), we observed that peak IL-1β was higher in those 18–35 years versus 60–69 years (*p* = 0.048; *p*_post-hoc_ = 0.043; Table 1), but no differences were detected for the IL-1β delta or AUC_i_ by age group. The TNF-α delta was greater in those 36–49 relative to 50–59 years old (*p* =  0.037; *p*_post-hoc_ = 0.030; Table 1). No other age group differences in postprandial inflammatory cytokine responses were observed.

**Table 1:** Inflammatory markers by age groups.

	Full Sample	18–35-year-olds	36–49-year-olds	50–59-year-olds	60–69-year-olds	
	(Max N = 55)	(Max N = 11)	(Max N = 14)	(Max N = 16)	(Max N = 15)	*P* value
**Inflammatory Markers**	M (SD)					
**IL-1β**						
* Fasting*	5.79 (4.90)	7.92 (8.17)	5.84 (3.38)	6.03 (4.65)	3.79 (1.91)	0.056
* Peak*	6.26 (4.74)	8.93 (8.45)^a^	6.68 (3.58)^ab^	5.78 (3.04)^ab^	4.28 (2.03)^b^	**0.048**
* Delta*	0.47 (1.42)	1.01 (1.47)	0.84 (0.92)	−0.25 (1.95)	0.50 (0.69)	0.281
* AUCi*	−1.06 (8.09)	−1.42 (12.20)	1.10 (3.98)	−3.62 (10.17)	−0.001 (2.79)	0.417

**IL-6**						
* Fasting*	5.76 (8.35)	7.41 (7.65)	8.37 (11.38)	2.47 (2.16)	5.38 (9.04)	0.252
* Peak*	7.58 (8.87)	7.62 (7.51)	10.63 (12.53)	4.62 (2.98)	7.67 (9.48)	0.912
* Delta*	1.83 (2.86)	0.21 (1.57)	2.26 (4.04)	2.15 (2.64)	2.29 (2.28)	0.078
* AUCi*^†^	2.20 (7.71)	−3.02 (9.66)^a^	2.79 (5.77)^a^	4.54 (8.92)^a^	3.15 (4.82)^a^	0.120

**IL-8**						
* Fasting*^†^	24.31 (28.91)	31.12 (29.41)^a^	34.54 (43.35)^a^	11.54 (10.19)^a^	22.53 (20.91)^a^	0.229
* Peak*	26.39 (30.17)	31.29 (29.19)	38.70 (45.75)	13.22 (10.40)	24.47 (22.13)	0.062
* Delta*	2.08 (4.47)	0.17 (4.12)	4.16 (6.88)	1.68 (2.83)	1.94 (2.38)	0.408
* AUCi*	1.30 (18.40)	−7.20 (24.38)	8.28 (23.56)	2.17 (12.18)	0.17 (10.45)	0.600

**IL-10**						
* Fasting*	21.51 (35.97)	25.61 (31.41)	33.23 (47.16)	18.30 (38.91)	11.76 (22.43)	0.084
* Peak*	22.31 (38.30)	25.78 (36.27)	33.54 (49.76)	18.57 (40.13)	13.44 (24.23)	0.198
* Delta*	0.40 (4.80)	0.17 (8.35)	0.31 (5.59)	0.27 (3.01)	0.78 (1.57)	0.430
* AUCi*^†^	−5.34 (23.55)	−8.91 (37.97)	−10.42 (30.26)	−3.98 (13.45)	0.64 (4.54)	0.571

**TNF-α**						
* Fasting*	18.96 (9.70)	19.24 (10.40)	19.97 (10.94)	20.12 (11.44)	16.57 (5.75)	0.757
* Peak*	18.91 (9.05)	18.83 (9.06)	21.44 (11.30)	18.32 (9.38)	17.24 (6.37)	0.678
* Delta*	−0.04 (3.52)	−0.41 (4.30)^ab^	1.47 (2.60)^b^	−1.80 (4.03)^a^	0.67 (2.41)^ab^	**0.037**
* AUCi*	−6.89 (17.94)	−8.50 (24.92)	−0.97 (12.48)	−14.26 (20.94)	−3.50 (10.78)	0.087

Data are presented as mean (SD). Log-transformed values used in all analyses except those denoted with ^†^, in which raw values were analyzed.

^†^Represents use of linear regression with age as a multicategorical predictor variable (reference: 18–35 years) in cases of non-normal outcome data after log transformation. *p* values represent results from one-way ANCOVA or linear regression adjusted for sex and BMI. *p* values < 0.05 were considered statistically significant. When *p* for an omnibus ANCOVA was < 0.05, Bonferroni’s test was performed as a post hoc test. Groups sharing the same letter (a or b) are not statistically different from one another based on post hoc analyses.

Abbreviations: IL interleukin; TNF tumor necrosis factor.

Lastly, linear regressions were performed to examine the continuous relationships of postprandial cytokines AUC_i_ and age, while considering BMI as a potential moderator and adjusting for sex. The overall models testing associations between age and IL-1β, IL-8, IL-10, and TNF-α responses were non-significant (*p*’s = 0.200−0.984; Table 2). A significant omnibus effect was found for IL-6 AUC_i_ (*p* = 0.017) and the overall model explained 21% of the variance in IL-6 responses. Specifically, older age was associated with a greater IL-6 response (*b* = 0.029, *p* = 0.010). No association between BMI and IL-6 AUC_i_ was observed (*b* = −0.040, *p* = 0.269); similarly, the interaction between age and BMI in association with IL-6 AUC_i_ was non-significant (*b* = −0.004, *p* = 0.105).

**Table 2:** Associations Between Age and Postprandial Inflammatory Response.

	IL-1β (n = 55)		IL-6 (n = 55)		IL-8 (n = 55)		IL-10 (n = 54)		TNF-α (n = 55)	
*Omnibus*	*R*^2^	*p*	*R*^2^	*p*	*R*^2^	*p*	*R*^2^	*p*	*R*^2^	*p*
	0.054	0.585	0.211	**0.017**	0.007	0.984	0.036	0.765	0.111	0.200
	***b***	***p***	***b***	***p***	***b***	***p***	***b***	***p***	***b***	***p***
Sex	−0.116	0.267	−0.624	0.054	0.040	0.764	−0.045	0.806	0.160	0.093
Age	−0.004	0.294	0.029	**0.010**	0.000	0.999	0.008	0.213	−0.003	0.396
BMI	−0.003	0.814	−0.040	0.269	−0.006	0.708	0.009	0.678	0.018	0.098
Age*BMI	0.000	0.850	−0.004	0.105	0.000	0.887	0.000	0.967	−0.001	0.220

Log-transformed values used in all analyses. Omnibus *p* values represent the overall regression model including sex, age, BMI, and age*BMI interaction in association with cytokine AUC_i_. Coefficient *b* values represent unstandardized regression coefficients for each predictor variable. Coefficient *p* values represent the association of each predictor variable with cytokine AUC_i_. *p* values < 0.05 were considered statistically significant.

Abbreviations: BMI body mass index; IL interleukin; TNF tumor necrosis factor.

## 4 Discussion

The aim of this work was to further characterize the cytokine response to high-fat meals in older adults by leveraging a relatively large sample and considering age as a continuous variable. We report that the high-fat meal increased IL-6, IL-1β, and IL-8 when analyzing all participants together. Further, we observed that age was positively associated with postprandial IL-6 AUC_i_ when examined continuously, but this effect was not apparent when dividing participants into age categories.

Repeated bouts of postprandial inflammation have been proposed as one contributor to cardiometabolic diseases, yet whether age is implicated in this response has been unclear. Mechanistically, it appears that increases in cytokines following high-fat meals are largely due to inflammation induced by unfavorable apolipoprotein profiles of triglyceride-rich lipoproteins (e.g., high apoC-III) and/or pro-inflammatory effects of saturated fatty acids liberated during triglyceride hydrolysis by lipoprotein lipase [9]. Our work partially resembles the broader literature on post-high fat meal changes in cytokines, but is somewhat in contrast to limited work in this field on older populations. For example, as in ∼70% of studies in generally healthy individuals, IL-6 increased after the high-fat meal across our full study sample [3]. Somewhat similarly, changes in IL-8 and IL-1β after high-fat meals have been documented in young-to-middle-aged adults; however IL-8 has only increased in ∼25% of studies [3,10], while IL-1β decreased in one instance and has otherwise been non-responsive to high-fat meals [3,11].

With respect to potential differences in postprandial inflammation with aging, a recent study reported decreased IL-10 after high-fat meal ingestion in adults ≥ 60 versus 18–35 years [5]. Although we did not observe changes in IL-10 generally or with increasing age, this finding is not entirely in opposition to our data. That is, a decrease in IL-10 – a cytokine commonly produced by anti-inflammatory T regulatory cells – would be expected to contribute to an overall pro-inflammatory tone similar to our observed increases in cytokines generally considered pro-inflammatory (i.e., IL-1β, IL-6, IL-8) [12]. The only other research on aging and postprandial inflammation to our knowledge was a relatively small study (n = 6–7/group) that observed no differences in IL-1β, IL-6, or TNF-α in adults aged 60–75 versus adults aged 20–25 [6]. Taken together, our work 1) agrees with a systematic review documenting that IL-6 increases following high-fat meals the majority of the time (32/45 studies) [3]; 2) adds to small, mixed literatures suggesting IL-8 and IL-1β may be altered by high-fat meal consumption (1/4 and 1/3 previous studies, respectively) [3]; and 3) provides evidence that the postprandial IL-6 response is greater with increasing age when examined continuously – a unique contribution of this work. Practical implications of these data may be that as people age, postprandial IL-6 excursions could contribute to observed low-grade inflammation in older adults, which may have relevance for chronic disease risk (e.g., CVD) [4]. However, more work is needed to confirm our findings and to determine if attenuating the IL-6 response to high-fat meals prevents chronic diseases with an immune component. From a study design standpoint, our data point to the need to consider age as a continuous variable when examining postprandial inflammation.

Weaknesses of the present report include that we were unable to measure postprandial cytokines more frequently than every two hours (e.g., hourly), so it is possible we did not capture every participant’s peak response, and the sample size within age subgroups were somewhat modest (*n*’s 11–16). Moreover, our study consisted of generally healthy individuals and therefore is not generalizable to other populations. Lastly, we can appreciate the limitations of the cross-sectional study design employed herein. Despite these limitations, this work leveraged a relatively large sample for a postprandial study in order to examine cytokine dynamics after a high-fat meal across the aging spectrum.

## 5 Conclusions

Overall, we provide evidence that increasing age is linked to a greater IL-6 response to a high-fat meal. Further, examining age continuously may have utility when studying aging and postprandial inflammation.

## CRediT authorship contribution statement

BHK: methodology, investigation, data curation, writing – original draft, writing – review and editing, visualization. NGK: formal analysis, data curation, writing – original draft, writing – review and editing, visualization. CMS: methodology, investigation, writing – reviewing and editing. ARM: methodology, investigation, writing – reviewing and editing. SEF: data curation, formal analysis, writing – review and editing. SRE: conceptualization, methodology, investigation, writing – review and editing, visualization, supervision, funding acquisition, project administration.

## Funding

This work was supported by the Oklahoma Center for the Advancement of Science and Technology (OCAST; HR20-027). The sponsor has no role in study design; in the collection, analysis and interpretation of data; in the writing of the report; and in the decision to submit the article for publication.

## Declaration of Generative AI and AI-assisted technologies in the writing process

The authors did not use AI when preparing this manuscript.

## Data statement

Data are available upon reasonable request.

## Declaration of competing interest

The authors declare none.
