# Supplementary material for: Postprandial inflammation across the aging spectrum
Source: J Nutr Health Aging. 2025 Jan 8;29(3):100468. doi: 10.1016/j.jnha.2024.100468 (PMC12179991; doi:10.1016/j.jnha.2024.100468)
Supplement: Supplementary file 1 [file mmc1.docx]

| **Table A1. Participant Characteristics** | | | | | | |
| --- | --- | --- | --- | --- | --- | --- |
|  | Full Sample  (max N = 56) | 18–35-year-olds  (Max N = 14) | 36–49-year-olds  (Max N = 14) | 50–59-year-olds  (Max N = 16) | 60–69-year-olds  Max N = 15 |  |
| *Demographics* |  |  |  |  |  |  |
| Age | 48 (14) | 27 (5) | 41 (4) | 53 (3) | 60 (3.3) |  |
| Sex (female) | 30 (53.6) | 7 (50.0) | 8 (57.1) | 10 (62.5) | 8 (53.3) |  |
|  |  |  |  |  |  |  |
| *Anthropometrics* |  |  |  |  |  |  |
| BMI (kg/m^2^) | 27.7 (4.3) | 27.2 (4.3) | 28.3 (4.3) | 27.0 (4.0) | 26.6 (4.7) |  |
| Body Fat (%) | 32.3 (8.3) | 29.4 (8.2) | 32.3 (8.7) | 35.9 (5.8) | 35.7 (3.6) |  |
| Lean Mass (%) | 64.7 (8.0) | 67.6 (8.0) | 64.5 (8.3) | 61.3 (5.5) | 49.4 (3.6) |  |
| Visceral Fat (g) | 520.0 (231.2) | 432.2 (199.3) | 461.1 (190.0) | 518.3 (252.6) | 595.2 (262.0) |  |
|  |  |  |  |  |  |  |
| *Metabolic Panel* |  |  |  |  |  |  |
| Total Cholesterol (mg/dL) | 187.0 (31.9) | 181.6 (46.4) | 183.3 (35.6) | 190.6 (24.2) | 180.0 (24.5) |  |
| HDL Cholesterol (mg/dL) | 58.9 (13.8) | 54.6 (10.6) | 60.4 (15.1) | 60.9 (14.6) | 55.5 (14.5) |  |
| LDL Cholesterol (mg/dL) | 106.5 (27.3) | 107.5 (40.1) | 100.4 (28.4) | 108.2 (22.7) | 104.1 (19.6) |  |
| nonHDL Cholesterol (mg/dL) | 127.6 (30.0) | 127.0 (43.3) | 122.9 (33.7) | 129.0 (25.7) | 124.4 (18.3) |  |
| VLDL Cholesterol (mg/dL) | 21.2 (7.8) | 19.7 (6.0) | 22.0 (11.6) | 22.2 (5.4) | 20.1 (8.1) |  |
| Triglycerides (mg/dL) | 104.9 (38.4) | 98.7 (30.2) | 112.2 (52.6) | 102.0 (27.8) | 100.7 (40.6) |  |
| Glucose (mg/dL) | 96.5 (7.5) | 91.0 (5.8) | 99.0 (7.9) | 96.2 (5.0) | 92.8 (8.9) |  |
| ALT (U/L) | 29.8 (12.3) | 27.9 (8.2) | 30.5 (13.4) | 30.4 (8.3) | 25.0 (7.7) |  |
| AST (U/L) | 30.9 (12.1) | 29.3 (5.5) | 30.4 (11.9) | 30.8 (5.3) | 25.0 (5.7) |  |
| **Table A1**. Data are presented as mean (SD) other than sex, which is shown as N (%). Abbreviations: **BMI b**ody mass index; **HDL** high-density lipoprotein; **LDL** low-density lipoprotein; **VLDL** very low-density lipoprotein; **ALT** alanine transaminase; **AST** aspartate aminotransaminase | | | | | | |
